# Lifestyle Practices and Obesity in Malaysian Adolescents

**DOI:** 10.3390/ijerph110605828

**Published:** 2014-05-30

**Authors:** Pey Sze Teo, Abdullah Nurul-Fadhilah, Mohd Ezane Aziz, Andrew P. Hills, Leng Huat Foo

**Affiliations:** 1 School of Health Sciences, Universiti Sains Malaysia, 16150 Kubang Kerian , Kelantan, Malaysia; E-Mails: peysze1985@msn.com (P.S.T.); nfha_9802@yahoo.com (A.N.-F.); 2 School of Medical Sciences, Universiti Sains Malaysia, 16150 Kubang Kerian, Kelantan, Malaysia; E-mail: drezane@gmail.com; 3 Centre for Nutrition and Exercise, Mater Research Institute, University of Queensland, South Brisbane, QLD 4101, Australia; E-Mail: andrew.hills@mater.uq.edu.au; 4 School of Biosciences, Taylor’s University, 47500 Subang Jaya, Selangor, Malaysia

**Keywords:** physical activity, sedentary practices, body fat, obesity, adolescents

## Abstract

*Aim:* To determine the influence of physical activity (PA) and sedentary behavior (SB) on obesity profiles of 454 Malaysian adolescents aged 12 to 19. *Methods:* Validated PA and SB questionnaires were used and body composition assessed using anthropometry and dual-energy X-ray absorptiometry (DXA). *Results:* Gender-specific multivariate analyses showed boys with high levels of total PA and moderate-to-vigorous physical activity (MVPA) exhibited significantly lower levels of total body fat, percent body fat and android fat mass compared with low PA and MVPA groups, after adjusting for potential confounders. Girls with high SB levels showed significantly higher BMI, waist circumference and DXA-derived body fat indices than those at lower SB level. Multiple logistic analyses indicated that boys with low levels of total PA and MVPA had significantly greater obesity risk, 3.0 (OR 3.0; 95% CI, 1.1–8.1; *p* < 0.05) and 3.8-fold (OR 3.8; 95% CI, 1.4–10.1; *p* < 0.01), respectively, than more active boys. Only in girls with high SB level was there a significantly increased risk of obesity, 2.9 times higher than girls at low SP levels (OR 2.8; 95% CI, 1.0–7.5; *p* < 0.05). *Conclusions:* The present findings indicate that higher PA duration and intensity reduced body fat and obesity risk while high screen-based sedentary behaviors significantly adversely influenced body fat mass, particularly amongst girls when the PA level was low.

## 1. Introduction

The prevalence of childhood obesity, which has increased dramatically over the past three decades in Malaysia and worldwide, is acknowledged as one of the most serious public health challenges of the 21st century [1]. Growing evidence indicates that childhood obesity, as determined by an excess accumulation of body fat, results in a wide range of health risks [1,2]. Moreover, if excess body fat gained during childhood and adolescence persists into adulthood there is an increased risk of developing chronic diseases in later life such as cardiovascular diseases, type-II diabetes, and certain cancers [2]. Thus, identification and prevention of obesity during adolescence is an important strategy to reduce present and future health risks.

Obesity results from a long-term energy imbalance, a combination of excess energy intake, low levels of energy expenditure and an inactive lifestyle [2,3]. The underlying causes of obesity during the growing years are variable with multi-factorial, non-modifiable and modifiable environmental factors involved [1,4] and potentially further complicated by biological growth factors during the rapid pubertal growth spurt [5]. Whilst a healthy diet and an active lifestyle are important strategies to prevent excessive weight gain and the risk of obesity [2,3], inactive lifestyle practices such as television-viewing, computer and media use has been shown to have profound effects on obesity risk among Caucasian populations [6,7]. However, the differential effects of physical activity (PA) and sedentary behavior practices on obesity levels in adolescents have yielded contradictory results. For example, a lifestyle intervention trial to reduce television-viewing and computer use was significantly associated with lower BMI in children, independent of total PA levels [6] and in Caucasian children aged 8–10, only moderate-to-vigorous physical activity (MVPA) was independently associated with body fat levels assessed by dual-energy X-ray absorptiometry (DXA). There was no significant association between sedentary practices and body fat levels in both genders [8]. Similarly, a lifestyle intervention with behavior modification and movement skills practice showed a profound effect on weight and body mass index (BMI) [9]. Some studies have also suggested that the relationship between PA, sedentary behavior (SB) and obesity appears to be gender-specific [10,11], possibly due to differences in body composition profiles during the pubertal growth spurt and differences in lifestyle practices.

To the best of our knowledge, no study has addressed these issues among Asian adolescents of diverse ethnic background. A better understanding of the lifestyle practices of Asian adolescents is particularly important based on reports of increasing inactivity in this age category [3,12]. Similarly, more definitive information regarding the relationships between PA, sedentary behavior practices and obesity could assist in the development of effective and innovative lifestyle intervention programs during the growing years. The aim of the present study was to determine the influence of lifestyle practices on body composition and body fat assessed by DXA among adolescents from diverse ethnic backgrounds in Malaysia.

## 2. Methods

This cross-sectional study was undertaken in the District of Kota Bharu, Kelantan, Malaysia. A convenience sample of 456 adolescents aged 12 to 19 was recruited. Recruitment was based on advertisements, school and community announcements, and peer-to-peer referral in community areas. Eligible participants were selected if they were healthy and physically active, having no clinical signs of bone-related disorder that could potentially prevent them from being physically active, and taking no medications known to influence bone metabolism. Two girls were excluded from the final analysis, one because she was unable to be scanned, and the other because regional body composition data were missing. In total, 454 adolescents comprising 204 boys and 250 girls of Malay and Chinese origin were included. The study was approved by the Research Human Ethics Committee of Universiti Sains Malaysia (USM). Written informed consent was obtained from both participants and parents or guardians prior to the study.

A validated self-administered past one-year PA questionnaire (PAQ) was used to assess the type, frequency and intensity of PA practiced by participants. Details of the validity and reproducibility of this PAQ have been described elsewhere [13]. In brief, the PAQ was used to collect both qualitative and quantitative information in three domains: school-based, leisure-based and household-based activities, including duration and intensity. Information on participation in organized sports either in school or in leisure time was also gathered. PA intensity categories: light (<3 METs), moderate (3 to <6 METs) and vigorous (≥6 METs) were employed according to the revised PA compendium [14]. Screen-based sedentary practices was estimated by total daily duration of SB. Participants were asked detailed information on weekly time spent in television-viewing, video games and computer and/or internet use including usual school day and a usual weekend day.

Details on assessment procedures for body composition and pubertal growth status have been reported elsewhere [15]. In brief, body weight, height, and waist circumference (WC) and hip circumference (HC) were assessed according to WHO standards. All measurements were taken twice, however if measurements differed by more than 1.0 cm or 1.0 kg, a third measurement was taken and mean of the two closest measurements recorded. Body composition profiles of participants was assessed using DXA (GE Lunar Prodigy, DPX; Lunar Corp., Madison, WI, USA) located at the Department of Medical Radiology, University Hospital and scans analyzed using software provided by the instrument manufacturer (enCORE software version 12.2). Total body fat (TBF, kg) and regional fat mass were also obtained. Pubertal growth was determined by a self-reported assessment of breast and pubic hair development for girls, and genital hair development for boys according to Tanner [16]. A validated qualitative past one-year food frequency questionnaire (FFQ) was used to assess dietary intake [17] with a comprehensive list of food photos and common household measures used to help quantify food items with the assistance of trained interviewers. Completed FFQs were then re-checked by a trained nutritionist for completeness and accuracy.

Data analyses were performed using the SPSS for Windows version 20.0 (SPSS Inc. Chicago, IL, USA) and a *p* < 0.05 was considered significant. BMI was converted to age- and sex-specific BMI *z*-scores using the Lambda-mu-sigma (LMS) method with the UK 1990 growth reference data [18]. BMI classifications were based on gender- and age-specific cut-off points recommended by the World Health Organization (WHO) [19]. Percent body fat (%BF) percentiles by age and gender were calculated using the LMS method [18]. There is no currently accepted gold standard for the definition of excess body fat in growing children and adolescents. However, ≥90th percentile of %BF has been shown to correspond with excess body fat in this population [20]. Given this precedent, the sex-specific and age-adjusted %BF of ≥90th percentile was used as the cut-off point to classify obesity in the present study. MVPA was derived from PA with metabolic intensity level equal to and greater than three METs. Descriptive statistics are reported as mean values ± SD, unless otherwise indicated. All variables were tested for normality using the Kolmogorov-Smirnov test prior to any statistical comparisons. Natural logarithmic transformations were applied for all skewed distributed variables (*i.e.*, PA profiles, SB time and dietary energy and fat density intake) prior to further statistical analyses. An independent student *t*-test was used to determine the differences of all variables between genders and ethnicity. Since there were significant between-gender differences in PA and body composition profiles, results are analyzed separately for each gender. A partial-adjusted correlation analysis was used to assess the magnitude of the association of total PA and MVPA levels and SB time on all body outcome parameters, after adjusting for age, pubertal growth status and ethnicity. Gender-specific multivariate analysis of covariance was used to determine the independent influence of total PA and MVPA level, and SB time on all body composition outcomes, by adjusting all known potential biological, socio-economic and dietary confounders such as age, pubertal growth status, ethnicity, household income, dietary energy and fat density intakes. Further, multiple logistic regression model analysis was performed to examine the odd ratios of obesity risk as assessed by %BF of °90th percentile according to age- and sex-specific by total PA, MVPA and SB levels, by further adjusting all confounders used as well as total PA and SP levels. Where appropriate, interaction between PA, and SB and body composition were also tested.

## 3. Results

Table 1 shows the general characteristics of socio-demographic, lifestyle behavior practices, dietary intake and body composition profiles for boys and girls by ethnicity. The mean age of participants was 15.3 ± 1.9 years, with the majority (72.5%) within the normal BMI range. Almost all participants (95.6%) had at pubertal stage of 2 to 5. Average daily time spent on PA and MVPA was 1.6 h (95% CI, 1.5–1.8 h) and 1.2 h (95% CI, 1.1–1.3 h), respectively, while mean daily duration spent on SP was 3.5 h (95% CI, 3.3–3.6 h). In general, adolescent boys had significantly higher daily PA levels (at least *p* < 0.01) and MVPA (all *p* < 0.001) compared to girls of similar ethnicity. In contrast, there was no significant between gender-difference in daily SB. As expected, adolescent boys had significantly higher body weight, height, WC, WHR and energy intake than girls. In contrast, girls had higher TBF (*p* < 0.001), and %BF (*p* < 0.001) than boys. There were no significant between gender differences in age, BMI and pubertal growth status.

Multivariate analysis of gender-specific models examined the independent influence of total PA, MVPA and SB levels on body composition and DXA-derived body fat indices, after full adjustment for age, pubertal growth status, ethnicity, household income and dietary energy and fat density intakes (Table 2). Boys with low MVPA exhibited significantly higher levels of TBF (*p* = 0.006), %BF (*p* < 0.001), and android fat mass (*p* = 0.005), compared to boys in the high MVPA group, after full adjustment of these covariates and a daily SB duration. A similar result was found for total PA where boys with higher levels (≥1.5 h) had significantly lower levels of DXA-derived body fat indices compared to boys with PA levels <1.5 h/day. No such difference was found for either frequency or intensity of daily PA in girls. However, when the association between body composition and SB was examined in both genders, there was only a significant association in adolescent girls. 

**Table 1:** General characteristics and body composition profiles (mean ± SD) of adolescent boys and girls by ethnicity, *n* = 454.

	Malay (*N* = 236)		Chinese (*N* = 218)	
	Boys (*N* = 104)	Girls (*N* = 132)	Boys (*N* = 100)	Girls (*N* = 118)
Age, years	15.4 ± 1.9	15.2 ± 1.9	15.2 ± 1.9	15.4 ± 1.9
Body weight, kg	52.5 ± 14.1	48.5 ± 13.4 ^*^	55.9 ± 15.0	51.2 ± 10.2 ^**^
Height, m	1.6 ± 0.1	1.5 ± 0.1 ^***^	1.6 ± 0.1	1.6 ± 0.1 ^***^
BMI, kg/m^2^	20.4 ± 4.3	20.6 ± 4.8	20.7 ± 4.2	21.1 ± 3.8
Thinness ^a^	9.6 (10)	10.6 (14)	6.0 (6)	5.1 (6)
Normal	70.2 (73)	72.0 (95)	75.0 (75)	72.9 (86)
Overweight and obese	20.2 (21)	17.4 (23)	19.0 (19)	22.0 (26)
BMI *z*-score	0.03 ± 1.36	−0.10 ± 1.44	0.20 ± 1.32	0.15 ± 1.24
Pubertal status ^a^				
Pre-pubertal	5.8 (6)	0.8 (1)	13.0 (13)	0.0 (0)
Pubertal	78.8 (82)	67.4 (89)	71.0 (71)	78.8 (93)
Post-pubertal	15.4 (16)	31.8 (42)	16.0 (16)	21.2 (25)
Dietary energy intake ^b,c^, kcal/day	2,408 (2,255–2,437)	2,178 ^**^ (2,058–2,246)	1,860 (1,792–1,970)	1,649 ^*^ (1,642–1,828)
Fat density ^b,c^, gMJ^-1^	7.4 (7.2–7.5)	7.8 ^**^ (7.6–7.9)	7.1 (6.8–7.3)	7.4^*^ (7.2–7.6)
Total PA ^b,c^, h/day	1.7 (1.8–2.4)	1.1 ^***^ (1.2–1.5)	1.4 (1.6–2.4)	0.8^**^ (1.0–1.5)
MVPA ^b,c^, h/day	1.3 (1.5–2.1)	0.4 ^***^ (0.5–0.8)	1.0 (1.4–2.1)	0.4^***^ (0.6–1.0)
Screen-based sedentary, SB ^b,c^, h/day	2.8 (2.8–3.6)	3.1 (3.0–3.6)	3.7 (3.3–4.0)	3.8 (3.3–4.0)
Waist circumference (WC), cm	68.0 ± 11.3	65.1 ± 10.3 ^*^	69.6 ± 12.6	65.7 ± 8.7 ^**^
Hip circumference, cm	85.1 ± 10.2	87.8 ± 10.4	85.1 ± 10.5	88.5 ± 8.5 ^*^
WHR	0.8 ± 0.1	0.7 ± 0.1 ^***^	0.8 ± 0.1	0.7 ± 0.1 ^***^
Body fat mass, kg	9.9 ± 8.7	16.3 ± 8.9 ^***^	11.3 ± 8.4	17.4 ± 6.9 ^***^
Body fat percent, %	17.1 ± 10.0	31.7 ± 8.4 ^***^	18.8 ± 9.4	32.8 ± 7.1 ^***^
Android fat mass, kg	0.8 ± 0.8	1.1 ± 0.7 ^**^	0.9 ± 0.8	1.3 ± 0.6 ^**^

Notes: BMI, body mass index; MVPA, moderate-to-vigorous activity; PA, physical activity; WHR, Waist-Hip Ratio Values are presented as mean ± SD unless otherwise noted. ^a ^% (N); ^b^ Median (95%, CI); ^c^ Analyses based on log-transformed value; Significantly different from boys of similar ethnicity at ^*^ *p* < 0.05, ^**^ *p* < 0.01 and ^***^ *p* < 0.001.

Girls with high SP (≥3.5 h a day) had significantly higher BMI (*p* < 0.05), WC (*p* < 0.05), TBF (*p* < 0.01), %BF (*p* < 0.05) and android fat mass levels (*p* < 0.05), after adjustment for age, pubertal growth status, ethnicity, household income, dietary intake of energy and fat and total PA levels. No significant difference was found in boys, except for android fat mass.

**Table 2:** Multivariate analyses of total daily PA, MVPA and SB time on body fatness profiles of adolescent boys and girls (adjusted mean, 95%, CI).

	Boys		*p* value	Girls		*p* value
	Low	High		Low	High	
	Total PA^1,2,3^, h/day					
*N*	99	105		181	69	
PA duration, hours/day	**<1.5 h**	**≥1.5 h**		**<1.5 h**	**≥1.5 h**	
BMI, kg/m^2^	21.0 (20.2; 21.8)	20.2 (19.4; 20.9)	0.149	21.0 (20.4; 21.6)	20.5 (19.5; 21.4)	0.382
BMI *z*-score	0.23 (−0.03; 0.48)	0.01 (−0.23; 0.26)	0.242	0.04 (−0.15; 0.23)	−0.04 (−0.35; 0.26)	0.654
WC, cm	69.7 (67.4; 71.9)	67.9 (65.7; 70.0)	0.263	65.7 (64.4; 67.0)	64.6 (62.4; 66.7)	0.370
WHR	0.8 (0.8; 0.8)	0.8 (0.8; 0.8)	0.219	0.7 (0.7; 0.8)	0.7 (0.7; 0.8)	0.572
Total body fat, kg	11.9 (10.3; 13.6)	9.3 (7.7; 10.9)	0.027	17.2 (16.1; 18.3)	15.9 (14.2; 17.7)	0.235
% body fat, %	20.0 (18.2; 21.9)	15.9 (14.2; 17.7)	0.002	32.6 (31.5; 33.6)	31.3 (29.6; 33.0)	0.219
Android fat mass, kg	1.0 (0.8; 1.1)	0.7 (0.6; 0.9)	0.023	1.2 (1.1; 1.3)	1.1 (1.0; 1.2)	0.190
**MVPA^2,3^, h/day**						
*N*	88	116		199	51	
MVPA duration, h/day	**<1 h**	**≥1 h**		**<1 h**	**≥1 h**	
BMI, kg/m^2^	21.2 (20.4; 22.1)	20.1 (19.3; 20.8)	0.051	21.0 (20.4; 21.5)	20.3 (19.2; 21.4)	0.310
BMI *z*-score	0.27 (−0.00; 0.54)	0.0030 (−0.23; 0.24)	0.153	0.048 (−0.13; 0.23)	−0.11 (−0.46; 0.25)	0.447
WC, cm	70.2 (67.8; 72.6)	67.6 (65.6; 69.7)	0.120	65.9 (64.6; 67.1)	63.5 (61.1; 66.0)	0.103
WHR	0.8 (0.8; 0.8)	0.8 (0.8; 0.8)	0.092	0.8 (0.7; 0.8)	0.7 (0.7; 0.7)	0.019
Total body fat, kg	12.5 (10.7; 14.2)	9.1 (7.6; 10.7)	0.006	17.1 (16.1; 18.2)	15.6 (13.6; 17.7)	0.200
% body fat, %	20.7 (18.7; 22.6)	15.8 (14.2; 17.5)	0.0001	32.5 (31.5; 33.5)	31.2 (29.2; 33.2)	0.267
Android fat mass, kg	1.0 (0.9; 1.2)	0.7 (0.6; 0.9)	0.005	1.2 (1.1; 1.3)	1.1 (0.9; 1.2)	0.191
**SB^2,4^, h/day**						
*N*	117	87		135	115	
SP duration, h/day	**<3.5 h**	**≥3.5 h**		**<3.5 h**	**≥3.5 h**	
BMI, kg/m^2^	20.2 (19.5; 20.9)	21.1 (20.2; 21.9)	0.126	20.2 (19.5; 20.9)	21.6 (20.8; 22.3)	0.013
BMI *z*-score	0.0010 (−0.23; 0.23)	0.28 (0.00; 0.55)	0.139	−0.13 (−0.35; 0.10)	0.18 (−0.06; 0.42)	0.070
WC, cm	67.4 (65.4; 69.4)	70.5 (68.2; 72.9)	0.052	64.0 (62.5; 65.5)	67.0 (65.4; 68.7)	0.010
WHR	0.8 (0.8; 0.8)	0.8 (0.8; 0.8)	0.067	0.7 (0.7; 0.8)	0.8 (0.7; 0.8)	0.125
Total Fat Mass, kg	9.7 (8.2; 11.2)	11.8 (10.0; 13.5)	0.083	15.5 (14.2; 16.7)	18.4 (17.0; 19.8)	0.003
% Body Fat, %	17.1 (15.4; 18.8)	19.0 (17.0; 21.0)	0.173	31.1 (29.9; 32.4)	33.5 (32.1; 34.8)	0.013
Android fat mass, kg	0.8 (0.6; 0.9)	1.0 (0.8; 1.1)	0.038	1.1 (1.0; 1.2)	1.3 (1.2; 1.4)	0.010

Notes: Values are presented as mean ± SEM; ^1^ Total physical activity duration was calculated after excluding staircase climbing and walking; ^2^ All data analyses were based on log-transformed values; ^3^ Adjusted for age (years), pubertal Tanner stage, ethnicity, household income, total daily energy intake (Kcal), fat density intakes (g/MJ) and total SP levels (h/day); ^4^ Adjusted for age (*years*), pubertal Tanner stage, ethnicity, household income, total daily energy intake (*Kcal*), fat density intakes (*g/MJ*) and total PA levels *(h/day).* Significant difference with low group with the Bonferroni’s adjustment at ^*^ *p* < 0.05, ^**^ *p* < 0.01 and ^***^ *p* < 0.001.

Table 3 shows odd ratios (OR) for associations between total PA, MVPA and SP levels and obesity risk. Adolescent boys with daily total PA levels of <1.5 h/day had a significantly greater risk of being obese (OR 3.0; 95% CI, 1.1–8.1; *p* < 0.05) than boys with higher daily total PA levels. A similar observation was found when PA intensity was used, boys whose daily MVPA levels were <1 h exhibited 4 times the risk of being obese (OR 3.8; 95% CI, 1.4–10.1; *p* < 0.01). Although there was no comparable link between PA measures and obesity risk in adolescent girls, there was a significant negative influence of daily sedentary practices and obesity risk in girls. A significant, three times greater likelihood of risk of being obese was found among girls with SB levels ≥3.5 h/day had approximately three times greater likelihood of being obese than girls with SB levels of <3.5 h/day (OR 2.8; 95% CI, 1.0–7.5; *p* < 0.05), after adjustment of biological, dietary and lifestyle confounders.

**Table 3:** Multivariate logistics models of odd ratios (OR) for being overweight or obese compared with normal weight based on total PA, MVPA and SB time groups based on genders.

	Boys		Girls	
	Odds ratio (95%, CI)	*p*-value	Odds ratio (95%, CI)	*p*-value
Prevalence of overweight and obese	13.2% (27/204)		10.8% (27/250)	
**Total PA^1^, h/day**				
Low	3.0 (1.1–8.1)	0.029	1.7 (0.6–5.0)	0.302
High	1.0 (referent)	--	1.0 (referent)	--
**MVPA^1^, h/day**				
Low	3.8 (1.4–10.1)	0.008	2.3 (0.7–7.8)	0.198
High	1.0 (referent)	--	1.0 (referent)	--
**SB^2^, h/day**				
Low	1.0 (referent)	--	1.0 (referent)	--
High	2.4 (0.9–6.3)	0.066	2.8 (1.0–7.5)	0.043

Notes: ^1^ Adjusted for age (years), pubertal Tanner status, ethnicity, household income, total energy intakes (Kcal/day) and fat density intakes (g/MJ) and total SP levels (h/day); ^2^ Adjusted for age (years), pubertal Tanner status, ethnicity, household income, total energy intakes (Kcal/day) and fat density intakes (g/MJ) and total PA levels (h/day).

## 4. Discussion

Adolescent boys in the present study were more active, with higher daily PA and MVPA levels and significantly lower body fat levels. Consistent with other studies in Caucasians, such participants had a 3.0 to 4 times lower risk of being obese, independent of sedentary lifestyle practices [10,21]. In a large sample of 2,094 European adolescents, those with higher MVPA levels (≥55 min/day) had significantly lowered risk of overweight and obesity compared with those of low MVPA levels [22]. In Caucasian children aged 8 to 10 years, those who did not accumulate ≥60 min/day of MVPA were 2.2 times more likely to be overweight or obese [8].

Along with low habitual levels of PA, there is emerging evidence that sedentary behaviors are an independent risk factor particularly in growing children and adolescents, including in Malaysia. Screen-based sedentary behaviors or activities of low intensity include the most common, television viewing and video game and internet use [12,23]. Such practices are becoming the ‘lifestyle of choice’ among growing children and adolescents, concomitant with precipitous declines in PA levels [24]. While escalating rates of childhood obesity worldwide can be attributed to such a dramatic increase in inactive lifestyle practices, there is a remarkable inconsistency in relationships between screen-based sedentary behaviors, body fat and obesity risk in children and adolescents [23,24]. Such inconsistency could be due to differences in assessment methods, outcome measures and potential confounding factors. Several studies have reported a significant inverse association between sedentary practices and body fat levels [24] and obesity risk [25,26], few have examined whether these associations are independent of PA levels [26,27]. In the present study, adolescent girls with higher daily SB levels had significantly greater body fat and three times higher risk of obesity compared to girls with lower daily SB. These influences remained significant after full adjustment for potential biological, dietary and daily PA levels, suggesting that higher SB levels were significantly associated with higher adiposity and the risk of obesity in girls, consistent with other studies of adolescents from France [26] and Brazil [28]. In the former study, increased weekly time spent in SB (≥22 h/week) was significantly associated with greater risk of being obese. There was no significant relationship between total duration spent in PA levels and obesity risk [26]. Similarly, children aged 7 to 10 y, children with ≥ 4 hours of daily SB had significantly increased risk of obesity (by 2-fold) [28]. Findings from these cross-sectional studies are supported by a longitudinal analysis of 106 children followed-up from preschool to early adolescence. Children with ≥3 hours of daily SB had significantly higher levels of body fat, assessed by skinfolds, after adjusting for baseline body fat, PA levels, dietary energy and fat intake and parental BMI status [27].

In the present study, gender-specific differences were found between lifestyle PA and screen-based sedentary practices, body fat and obesity risk however, the significant influence of PA was only found among adolescent boys. The precise mechanism for this discrepancy is unclear however could be attributed to differences in duration and intensity of PA. Adolescent boys were more physically active with significantly higher total and intensity PA levels compared to girls, consistent with several previous studies [10,21]. These results suggest that body fat development might be more sensitive to variation in PA in boys compared to girls. In contrast, a high SB level was only significantly and positively associated with higher body fat level and greater risk of obesity in girls however, we found no relation between PA and body fat and obesity risk in girls.

A significant negative association between PA, body fat level and obesity risk was stronger in boys than in girls, consistent with other studies among Caucasian children and adolescents [10,29]. Must and her colleagues have suggested that the energy intake side of the energy balance equation may be more important in girls than boys [30] and PA may also have a stronger effect on appetite and satiety in boys and/or it may be that girls more frequently use dietary restraint to control body weight compared to boys [29]. The profound influence of SB’s on body fat in girls is consistent with Boone and colleagues who suggested a gender difference may be due to biological or metabolic changes in energy expenditure in response to screen time [25].

Some limitations of the present study must be acknowledged. First, as the study was cross-sectional, causality regarding the relationships between both PA and SB levels and body fat cannot be established. Secondly, self-administrated PAQs are subjective and associated with recall bias despite the instrument being validated in this population. However, the present PAQ assessment tool has been validated for the estimation of the habitual PA levels for the past one year among these adolescents, by taking into account of differences in sports events and seasonal variations of sporting events across a one year period. Furthermore, results may not be generalizable to all other Asian populations. Nevertheless, the study has several strengths including a large sample size and wide age range. Comprehensive body composition data were derived from DXA and the inclusion of important biological, dietary and lifestyle factors such as pubertal growth status, socio-demographic, dietary energy and fat intake and lifestyle practices in multivariate analysis model are further strengths of this study.

## 5. Conclusions

The main findings of the present study indicated that higher PA duration and intensity is significantly associated with lower body fat and obesity risk while high screen-based sedentary behaviors have a significant adverse influence, especially when PA was low, and particularly amongst girls. Effective intervention strategies should emphasize the promotion of healthy and active lifestyles among school-aged adolescents.
